# Attitudes and Behaviors Regarding Sun Exposure of Spain Population

**DOI:** 10.1111/phpp.70035

**Published:** 2025-07-03

**Authors:** Charles Taieb, Alba Crespo‐Cruz, Marketa Saint Aroman, Nuria Perez‐Cullell, Catherine Baissac, Bruno Halioua, Magdalena de Troya‐Martín

**Affiliations:** ^1^ Patients Priority Department European Market Maintenance Assessment Paris France; ^2^ Pierre Fabre Laboratories Barcelona Spain; ^3^ Pierre Fabre Laboratories Toulouse France; ^4^ Société Française des Sciences Humaines pour la Peau, Maison de la dermatologie Dermatologist Paris France; ^5^ Departament of Dermatology Hospital Costa del Sol Marbella Spain

**Keywords:** public health, solar exposure, Spain

To the Editor,

Exposure to solar ultraviolet radiation (UVR) is a well‐established primary risk factor for nonmelanoma skin cancer and melanoma. In Spain, a country in Europe enjoying around 3000 h of sunshine a year, the annual standardized mortality rate for melanoma was 2.10/100,000 inhabitants over the last known period 2000–2019, while for melanoma and nonmelanoma skin cancer, it was 1.28/100,000 [1]. Numerous public health actions have been undertaken to change sun protection behaviors and attitudes in Spain to reduce sun exposure and to prevent skin cancer.

In a 2014 study with Spanish students, 60.7% said they were aware of the harmful effects of the sun on their skin [2]. In recent years, numerous public health actions have been carried out in Spain to change sun protection behaviors and attitudes in order to reduce sun exposure and prevent skin cancer.

ALL (All Skins, All Colors, All Dermatoses) is a multinational, cross‐sectional study conducted in 20 countries using a web‐based online survey by a polling company (HC Conseil Paris, France) between January and April 2023. Institutional review board approvals were not required since the study did not involve any clinical examination and used anonymized data. The overall results have been published [3]. In this study, the results in the Spanish population are examined in further detail. For each country, a representative sample of the population aged 16 and over was constructed using the usual quota method (age, gender, geographical location, socio‐cultural level and phototype according to the Fitzpatrick classification). Fitzpatrick skin types were dichotomized based on susceptibility to ultraviolet‐induced erythema, with types I–III categorized as more susceptible to burning [FT I–III] and types IV–VI as less susceptible [FT IV–VI] [4, 5].

The aim of this publication was to investigate the level of awareness of the risks related to sun exposure, attitude toward sun protection, and sun protection behavior in the representative Spain population.

Since the study used anonymized data and did not involve any clinical examination, institutional ethics committee approvals were not required.

The questionnaire gathered information about the individuals' demographic and socio‐demographic profiles. The individuals were asked to specify the times of exposure to the sun, the notion of knowledge of the recommendations of the public health experts who advise against exposure between 11 a.m. and 4 p.m., the reasons for which they were exposed to the sun in that time range, and the use or non‐use of sunscreen products. It was asked to specify the motivations provided by sunscreen users and the justifications given by non‐users. Descriptive analyses using absolute and percentage frequencies were performed.

A population of 4000 was selected, including 1965 (49.1%) males and 2035 (50.9%) females, with a mean age of 44.84+/−15.14 (16–90 years). About 83.8% (3354/4000) were identified as more susceptible to burning according to the Fitzpatrick classification.

About 23.1% of respondents reported no sun exposure, 23.9% of FT I–III people vs. 18.7% of FT IV–VI people (*p* < 0.005). About 27.6% stated that they had preferred sun exposure before 11 a.m., and 28.3% exclusively after 4 p.m., while 1896 (47.4%) respondents admitted to having been exposed to the sun between 11 a.m. and 4 p.m. The most frequent reason for this risky behavior was that it corresponded to the most convenient hours for their activities (47.0%) and when they were available to do so (48.2%) (Table 1).

**TABLE 1:** Reasons given by people who expose themselves to the sun between 11 a.m. and 4 p.m. and who say they are aware of the public health recommendations (*n* = 1508).

	*n*	%
I don't understand	61	4.0%
These are the most convenient times of the day for my activities	709	47.0%
These are the most pleasant hours of the day	425	28.2%
These are the hours when I am available	727	48.2%
I don't believe in prevention messages	77	5.1%

About 80.3% of the respondents declared being aware of public health experts' recommendations against exposure between 11 a.m. and 4 p.m. Those who exposed themselves in that time interval were as well informed of these recommendations as those who were not (79.5% vs. 80.9%, p 0.85). Among the respondents, 2549/3077 (82.8%) used sunscreen products during the sunniest period (39.7% every two hours). The motivation of those who use sun protection products was mainly the desire to avoid sunburn (67.6%) and to reduce the risk of skin cancer (57.4%) (Table 2). On the other hand, the 528 (17.2%) respondents who stated they did not apply sunscreen products explained their behavior mainly because they consider topical application tedious (43.6%) and because they do not think about it (31.9%).

**TABLE 2:** Motivations of users of sun protection products and justifications of non‐users, as recommended by public health guidelines.

		*n*	%
Rationale for respondents who use sunscreen products *n* = 2549	To avoid sunburn	1722	67.6%
	To spend more time in the sun	410	16.1%
	Because of a history of sunburns	447	17.5%
	As protection against accelerated skin aging	1177	46.2%
	As protection against the risk of skin tumors	1462	57.4%
	For other reasons	81	3.2%
Justification for respondents who do not use sun protection products as recommended by public health guidelines *n* = 1856	The products are too expensive	353	19%
	I don't think about it	592	31.9%
	I don't think it is useful	182	9.8%
	It is too tedious	809	43.6%
	I didn't know about this recommendation	217	11.7%

This study assesses both sun exposure risk behaviors and sunscreen use in the Spanish population. Despite the widespread dissemination of public health messages about the importance of sun protection, almost 47.4% of Spanish adults do not avoid sun exposure between 11 a.m. and 4 p.m. Furthermore, although the majority use sunscreens, there are still people who do not use them for various reasons (the price of the products, they think it is not useful, or they say they do not think about it) Table 2.

These findings highlight the need to implement more relevant educational measures targeting both the general population and, in particular, individuals at high risk of skin cancer, in order to persuade them to modify their behaviors. This includes avoiding sun exposure during peak radiation hours and promoting the effective use of sun protection. It appears essential that health authorities and professionals (such as doctors and pharmacists) collaborate to develop more pertinent and specific methods to enhance prevention efforts.

## Ethics Statement

This survey has been carried out according to the ICC/ESOMAR code of conduct.

## Conflicts of Interest

Charles Taieb was compensated for rewriting this article. Alba Crespo‐Cruz, Marketa Saint Aroman, Nuria Pérez‐Cullell, and Catherine Baissac are employees of Pierre Fabre. Bruno Halioua has no conflicts of interest in this project. Magdalena de Troya‐Martín has no conflict of interest.

## Data Availability

The data that support the findings of this study are available on request from the corresponding author. The data are not publicly available due to privacy or ethical restrictions.
